# Refractive errors and associated factors among patients visiting BoruMeda Hospital’s secondary eye Unit in Dessie Town, South Wollo Zone, Ethiopia

**DOI:** 10.1186/s12886-022-02539-z

**Published:** 2022-07-19

**Authors:** Bersabeh Besufikad, Wasihun Hailemichael, Lehulu Tilahun, Wondwossen Yimam, Samuel Anteneh

**Affiliations:** 1 Ophthalmology Department, Zewuditu Memorial Hospital, Addis Ababa, Ethiopia; 2 Department of Ophthalmology, Mizan Aman Hospital, Mizan Aman, Ethiopia; 3 Department of Emergency and Ophthalmic Nursing, Wollo University, Dessie, Ethiopia; 4 Department of Comprehensive Nursing, Wollo University, Dessie, Ethiopia; 5 Department of Adult Health, Wollo University, Dessie, Ethiopia

**Keywords:** Refraction, Refractive error, Associated parameters

## Abstract

**Background:**

In Ethiopia, refractive error is the second leading cause of vision impairment and the third main cause of blindness. Because refraction services are scarce and difficult to obtain, many people with refractive error live with impaired vision or blindness for the rest of their lives.

**Objective:**

The primary goal of this study was to determine the prevalence of refractive errors and associated factors among patients who visited Boru Meda Hospital.

**Methods:**

A retrospective cross-sectional study was conducted from April to June 2018, all patients who visited Boru Meda Hospital's secondary eye unit were deemed our source population; the sample frame was the secondary eye unit outpatient departments register. To select samples, simple random sampling was used.

Data was entered by using Epi-data version 3 and analysed with Statistical Package for Social Science 20. Tables and graphs were used to display descriptive statistics, and logistic regression was used to examine the relationship between the dependent and independent variables. At *p* < 0.05, statistical significance was inferred.

**Results:**

Refractive error was detected in 42 (18.3%) of study participants. The average age was 46.69 ± 20.77. There were 136 men and 93 women in this group (59.4 and 40.6%, respectively). Myopia was the most frequent refractive defect, accounting for 52.4% of all cases.

**Conclusion & recommendation:**

Refractive error is a widespread problem in our study area that affects people of all age groups. We recommend patients to have screening on regular basis so that refractive anomalies can be detected early.

## Introduction

Refractive error (RE) is a phenomenon that happens when the eye fails to concentrate light rays from objects onto the retinal plane, resulting in fuzzy images. Myopia (short sightedness), hyperopia (long sightedness), and astigmatism (no single point of focus in the eye) are the three types of refractive defects. Anisomery is a condition in which the refraction powers of two eyes differ [1].

Refractive error is one of the most common causes of vision impairment, accounting for 47% of all cases of vision impairment in high-income nations. In developing countries, refractive error has a substantial impact, perhaps resulting in decreased economic production [2].

Refractive error affects people's lives, whether they are children or adults, causing difficulties in performing regular tasks, decreasing their vision, and eventually causing blindness. It affects people of all ages, but the impact is thought to be greater in youngsters due to the longer delay. Nuclear sclerosis is the primary cause of refractive error in adults, which shows an increasing tendency with increasing sclerosis but reduces after compensating for it [2–4].

The right- to- site initiative, Vision 2020, was founded in 1999 with the goal of eliminating avoidable blindness by prioritizing a few particular causes of vision impairment and blindness based on their distribution, impact on the community, management potential, and affordability. One of the five priority issues addressed is refractive error. According to the most recent global estimates, 12.8 million children between the ages of 9 and 15 suffer from refractive error-related visual impairment [5].

Children are reported to be the most vulnerable segment of the population, with many suffering from vision impairment throughout their lives. Refractive error has gotten a lot of attention in the last two decades, with school-aged children being at a larger risk than the rest of the population [5]. Instead, they try to compensate for their vision problems by sitting close to the blackboard, pinching their eyes, and even avoiding work that requires good vision [6].

According to a national blindness survey conducted in Ethiopia in 2006, refractive error was found to be the second leading cause of visual impairment, accounting for 33.4%, behind cataract (42.3%), and the third leading cause of blindness, accounting for 49.9, 11.5, and 7.8%, following cataract and trachomatous corneal opacity respectively. Females were found to have higher rates of blindness and low vision than males, with 1.9 versus 1.2 for blindness and 4.1 and 3.1 for impaired vision, respectively [7].

Despite the fact that refraction management is relatively simple and inexpensive with spectacles, millions of children and adults are dropping out of school and their productivity is declining. Refractive error is one of the most common eye diseases related to regular absenteeism and poor productivity, according to studies conducted in Nigerian hospitals and industries [6].

As previously stated, refractive error is a problem that requires attention, but no such attention has yet been given in the community. In addition, as per the knowledge of investigators, there were few studies in Ethiopia and no research conducted specifically at study area where it’s the main center of ophthalmic health in the region. As a result, we are interested in studying the burden and encouraging stakeholders to work on it in order to alleviate the problem.

## Methods and materials

Boru Meda Hospital is one of seven hospitals in the South Wollo zone, located 401 km from Addis Ababa and only seven kilometers from Dessie, the zone's capital. The hospital is well-known for providing eye care to a population of 1.2 million people living within its catchment region. Ophthalmic professionals of all levels, including ophthalmologists, ophthalmic officers, optometrists, and nurses, are available at BoruMeda Secondary Eye Unit. The secondary eye unit provides eye care to patients by examining and treatment of eye infections, surgery of cataract, glaucoma and trichiasis are provided in the secondary eye unit.

From April to June 2018, a hospital-based retrospective cross-sectional study was done using secondary data from registration books and patient cards. The investigation was conducted from April to Jun 2018, at Boru Meda Hospital Secondary Eye Unit. All patients that visited Boru Meda Hospital Secondary Eye Unit were considered a source population. All patients that visited BoruMeda Hospital Secondary Eye Unit during the study period were included in the study population.

All patients who visited the Boru Meda hospital's eye-out patient department for an ocular ailment between April and June 2018 and registration book and medical cards with recorded needed variables were included in the study. Patients with uncorrected RE or amblyopia who visited the eye unit between April and June 2018 and incomplete registration book and medical cards were excluded from the research.

A single population proportion calculation was used to calculate sample size. As a result, 229 patients were randomly selected for the study using a simple random sampling technique from the registration book. That is: -

$$\mathrm n=\frac{{\lbrack{\mathrm Z}_{\mathrm a2}\rbrack}^2\ast\mathrm P\ast(1-\mathrm P)}{{\mathrm d^2}_{}}$$

plus 10% non-response rate where

n = the final sample size

p = proportion/prevalence of myopia, which is 16.6% [8]

d = maximum allowable error in this case 0.05(5%)

Accordingly the sample size for this study = (1.96)^2^0.1606(1–0.1606)/ (0.05^2^)

 = 3.8416*0.13480764/0.0025 = 208

Plus 10% non^−^response rate

n = 229

Ophthalmic nurses working in the secondary eye unit used a WHO standard check list to collect data from the registration book and patient cards from April to June 2018. To ensure data quality, data collectors received a half-day training on the data collecting instrument. Pretest was done on 10% of the overall sample size (23 samples) in Dessie referral hospital. Data was collected by ophthalmic nurses, and the quality of the data was reviewed on a daily basis by principal investigators. The questions were evaluated for clarity, completeness, and consistency.

The data was entered into Epi data version 3 and then transferred to SPSS V.20. Descriptive statistics such as frequency distribution, table, graph, and summary measures were produced to explain the research population in connection to pertinent variables. Furthermore, each independent variable to the outcome variable was subjected to a bivariate binary logistic regression analysis to discover statistically significant associated factors. In multivariate binary logistic regressions, variables with a *P*-value of less than 0.05 were considered statistically significant and were given a 95% confidence interval and an AOR.

## Result

### Socio-demographic characteristics of respondents

For the study, a total of 229 case records were obtained. The records of 229 participants were reviewed. There were 136 men and 93 females aged 1–100 years (59.4 and 40.6%, respectively). The subjects' average age was 46.69 ± 20.770 years. The majority of the cases (162 (99 males and 63 females) live in the rural region (70.7%), while 67 (37 males and 30 females) dwell in the urban area (29.3%) (Table 1).

**Table 1:** Shows the cross tabulation age and gender distribution of patients visiting BoruMeda Hospital's secondary eye unit in Dessie Town, South Wollo Zone, Ethiopia (*N* = 229)

**Age of participant**	**Sex of participant**		**Frequency (Percent)**
	Male	Female	
< 10	4	4	8 (3.5)
11–20	12	12	24(10.5)
21–30	15	16	31(13.5)
31–40	12	16	28(12.2)
41–50	17	14	31(13.5)
51–60	33	14	47(20.5)
> 60	43	17	60,926.2)
**Sex of participant**	**Address of participant**		
	Urban	Rural	
Male	37	99	136 (59)
Female	30	63	93(40.6)

### Magnitude of refractive error

Refractive error was identified in 42 of the 229 study participants (18.34%), 95%C.I (15.6-22.47%). Two-thirds (66.66%) of the 42 people with refractive error were females, and one-third (33.33%) were men. Myopia was detected in 22 (52.4%) people; astigmatism in 12 (28.5%) people, and hyperopia in just 8 people (19%). Myopia was more common in women, while astigmatism was more common in men. Almost two-thirds of the refractive error distribution was seen in those aged 11 to 30 years (40.5%) and 51 to 60 years (31%) (Table 2).

**Table 2:** Shows the distribution of age and refractive error in patients visiting BoruMeda Hospital's secondary eye unit in Dessie Town, South Wollo Zone, Ethiopia (*N* = 229)

Age category	Type of refractive error			Total	%
	myopia	hyperopia	Astigmatism		
1–10	0	0	O	0	0
11–20	6	0	2	8	19%
21–30	8	1	2	11	26.2%
31–40	2	2	2	6	14.3%
41–50	0	1	3	4	9.5%
51–60	5	3	3	11	26.2%
> 60	1	1	0	2	4.8%
Total	22	8	12	42	100%

### Refractive error degree

In this study, the degree of refractive error in myopic individuals (22 cases) was 5.2%, which is about half of the total (12 cases) only 4.4% of them have > -3D, while nearly half of the remaining (10 instances) have 3D. On the other hand, three out of every eight instances with hyperopia have > 3D, and one out of every eight cases has 3D. Astigmatism accounts for 5.2% of all refractive cases (Table 3).

**Table 3:** Shows the distribution of refractive error degrees of patients visiting BoruMeda Hospital's secondary eye unit in Dessie Town, South Wollo Zone, Ethiopia (*N* = 229)

Degree of refractive error	Frequency	Percent
Normal	187	81.7
Myopia (> -3D)	12	5.2
Myopia(<—3D)	10	4.4
Hyperopia (> 3D)	2	.9
Hyperopia (< 3D)	6	2.6
Astigmatism	12	5.2
Total	229	100.0

### Presenting visual acuity and Medical history

The distribution of presenting visual acuity from 229 frequencies, with about half of the cases falling into this category.

One hundred fourteen people (49.7%) are normal; nearly a quarter (47%) have moderate visual impairment, and 21 people (9.2%) have severe visual impairment. The remaining 41 cases (17.9%) were blind.

The vast majority of the study population, 214 (93.4%), had no medical history; however, 15 (6.6%) had co-morbid medical history, with 7 having diabetes and the other 7 having hypertension (Table 4).

**Table 4:** Medical history of patients visiting BoruMeda Hospital's secondary eye unit in Dessie Town, South Wollo Zone, Ethiopia (*N* = 229)

Variables		Frequency	Percent
Medical History	Have medical history	15	6.6
	Have no medical history	214	93.4
If Yes, type of medical history	DM	7	3.1
	Hypertension	7	3.1
	Other	1	.4

### Factors associated with refractive error

On bivariate regression, sex, age category, medical history, and surgical history show a significant *p*-value of less than 0.3 (CI of 95% and adjusted odd ratio) for dependent variables from other nondependent factors.

In multivariate binary logistic regressions, variables having a *P*-value of less than 0.25 in bivariate analysis were imported. In multivariate binary logistic regressions, those factors with *P*-value less than 0.05 provided with 95% CI and AOR sex, medical history, and surgical history are statistically significant variables. Females had a four times higher risk of developing refractive error than males. The patient's medical history (diabetes, hypertension) is strongly associated with refractive error (Table 5).

**Table 5:** Multivariate regression among patients visiting BoruMeda Hospital's secondary eye unit in Dessie Town, South Wollo Zone, Ethiopia (*N* = 229)

	COR	AOR (95% CI)	*P*-value
Sex			
Male^ref^			
Female	3.75	3.9 [1.75,8.87]	0.001*
Age	0.974	0.76 [0.36, 1.84]	0.125
Medical history			
Yes	2.39	5.57 [1.12,27.7]	0.036*
No^ref^			
Surgical history			
Yes	0.077	2.5 [0.01, 64]	0.017*
No^ref^			

(ref- reference), * statistically significant

## Discussion

In this study, we discovered that refractive error was responsible for (18.34%), 95%C.I (15.6-22.47%) of the eye problems encountered in our research area. This is consistent with recent data from other Nigerian eye-care facilities, which indicated that refractive error ranged between 18.6 and 22.0% [9]. This finding was lower than hospital-based research in Ghana, which found refractive error in 44.3% of the subjects [6], and also lower than community-based investigations in Ethiopia [6, 10, 11]. This implies a great effort is needed by policy makers and the patients themselves to reduce the issue. However, it's worth noting that the rate reported by this hospital-based study is higher than several other community-based studies in Nigeria, which have shown rates ranging from 2.6 to 15.4% [9, 12]. This indicates that uncorrected or under-corrected refractive errors have severe consequences for the individual, family and society. These include lost educational and employment opportunities, as well as economic costs to the family and government and generally impair quality of life.

Females were almost four times more affected than males in this survey. This occurrence was observed across all age groups. The larger female population is consistent with findings from previous research in developing countries, such as Nigeria [9, 12]. This study's female gender preponderance could be explained by the fact that specific forms of refractive errors are more common in females than in males.

Myopia was the most common eye condition in this investigation. This accounted for 52.4% of all refractive error cases. The most prevalent refractive error in Africa has a variety of reports. While myopia has been identified as the most prevalent distance refractive defect, astigmatism [13] and hyperopia [12, 14, 15] have also been identified. Uncorrected distance refractive error (mostly myopia) has been identified as the leading cause of vision impairment worldwide, and this trend is expected to continue. This result is similar to Adegbehingbe et al. in Ile-Ife (22.7%) [13] and Emerole et al. in Owerri (23.4%) in similar hospital-based investigations, as well as 26.99% in population research in Southern India, but lower than Adeoti and Egbewale's findings in Osobgo (39.2%) [15].

In comparison to males, more women [16] had myopia in varied degrees [4]. In an epidemiological review of myopia, women were found to have a higher prevalence than men. Myopia was at its peak between the ages of 10 and 30. The severity of myopia varies with age, with the majority of cases occurring between the ages of one and ten years and remaining relatively stable between the ages of twelve and fifty years [17].

### Limitation

One disadvantage of this study is that it was conducted in a hospital setting, which could lead to an overestimation of the magnitude of refractive error because most people go to the hospital for vision problems. Regardless, because the hospital is a community-oriented health care provider, the findings can still be projected to the community.

## Conclusion

As a conclusion, refractive error is more prevalent in this study area that affects people of all ages. Also, myopia is the most frequent kind of refractive error, and astigmatism affects a large percentage of patients. Variables like sex, medical history, and surgical history were proved to be statistically significant with refractive error. As a result, we urge that they get screened on a regular basis so that refractive anomalies can be detected early. In addition, we recommend hospital, staffs and clients to make a big concern for patients with past history of medical and surgical cases since they are associated with eye anomaly. The staff will make every effort to improve and/or adjust the situation.

## Data Availability

The datasets generated and/or analyzed during the current study are not publicly available due to privacy need of data set, but are available from the corresponding author on reasonable request.
